# A truncated mutation of MucA in *Pseudomonas aeruginosa* from a bronchiectasis patient affects T3SS expression and inflammasome activation: Effect of a truncated mutation of MucA on T3SS expression and inflammasome activation

**DOI:** 10.3724/abbs.2022169

**Published:** 2022-11-24

**Authors:** Yanan Liu, Lin Du, Yongzhang Zhu, Xuefei Liu, Ning Zhou, Congcong Li, Qingtian Li, Ping He

**Affiliations:** 1 Department of Laboratory Medicine Ruijin Hospital Shanghai Jiao Tong University School of Medicine Shanghai 200000 China; 2 Department of Medical Microbiology and Parasitology Shanghai Jiao Tong University School of Medicine Shanghai 200000 China; 3 School of Global Health Shanghai Jiao Tong Univerisy School of Medicine & Chinese Center for Tropical Diseases Research Shanghai 200000 China; 4 Department of Respiratory and Critical Care Medicine Ruijin Hospital Shanghai Jiao Tong University School of Medicine Shanghai 200000 China

**Keywords:** MucA, bronchiectasis, Type III secretion systems, inflammasome

## Abstract

*Pseudomonas aeruginosa* is an opportunistic pathogen that causes chronic airway infection in bronchiectasis patients and is closely associated with poor prognosis. Strains isolated from chronically infected patients typically have a mucoid phenotype due to the overproduction of alginate. In this study, we isolate a *P*. *aeruginosa* strain from the sputum of a patient with bronchiectasis and find that a truncated mutation occurred in *mucA*, which is named *mucA117*. *mucA117* causes the strain to transform into a mucoid phenotype, downregulates the expression of T3SS and inflammasome ligands such as *fliC* and allows it to avoid inflammasome activation. The truncated mutation of the MucA protein may help *P*. *aeruginosa* escape clearance by the immune system, enabling long-term colonization.

## Introduction

*Pseudomonas aeruginosa* is an opportunistic pathogen that can cause acute and chronic infections in humans, particularly in patients with compromised immune defense. Acute infections usually include respiratory and urinary tract infections, such as ventilator-associated pneumonia [1]. Chronic infections usually occur in patients with cystic fibrosis (CF), chronic obstructive pulmonary disease (COPD) and bronchiectasis [ 2, 3] . During chronic infection, *P*. *aeruginosa* is difficult to eliminate, which will cause irreversible and destructive changes in the respiratory system and have a serious influence on the patient’s quality of life and survival time [4].

Bronchiectasis is a chronic endobronchial suppurative disease that is characterized by damage to the bronchi due to repeated infections of various microorganisms and inflammation of the airways, resulting in irreversible expansion of the bronchi [ 5, 6] . In the Asia-Pacific region, the prevalence of bronchiectasis is high, and the potential genetic predisposition may be one of the reasons for its high prevalence. *P*. *aeruginosa* is one of the most common pathogens isolated from the sputum of patients with bronchiectasis, regardless of whether it is in stable or acutely worsening periods [7]. At present, chronic colonization by *P*. *aeruginosa* is an important risk factor for the severity and prognosis of bronchiectasis and has been included in various bronchiectasis severity scoring systems, such as BSI [8] and FACED score [9].

During chronic lung infections, common genetic adaptations and advantageous phenotypic switches occur in *P*. *aeruginosa* to ensure its persistence in the lung [10]. These adaptation processes include conversion to the mucoid phenotype [11], avoidance of inflammasome activation [12], loss of motility [13], inactivation of quorum sensing function [14], and so on. Mucoid colonies, which are caused by overproduction of the polysaccharide alginate, are widely considered to be a marker for the transition to chronic infection [15] and can be used as an independent predictor to identify poor prognosis with *P*. *aeruginosa* pneumonia [16]. *P*. *aeruginosa* transforms into a mucoid phenotype, usually mediated by the destruction of *mucA*. Mutations of *mucA* are found in 82% of mucoid isolates of CF patients [17]. However, there are few reports on *mucA* mutations in bronchiectasis. The binding between MucA and AlgU prevents AlgU from encoding the enzymes required for alginate synthesis. Loss-of-function mutations in MucA lead to elevated transcription of the *alg* operon and result in alginate overproduction. *P*. *aeruginosa* triggers robust inflammatory responses during acute infection, which usually leads to pathogen clearance and resolution of infection [18]. However, during chronic pulmonary infections, these phenotypic switches help the bacteria avoid inflammasome activation and phagocytic clearance, therefore enhancing their ability to persist [19].

We currently know little about *mucA* mutations of *P*. *aeruginosa* isolated from patients with bronchiectasis. In this study, we isolated *P*. *aeruginosa* strain L012 from the respiratory tract of a patient with bronchiectasis and found that it showed mucoid colonies and weakened cytotoxicity compared with the wild-type strain PAO1. Through comparative analysis of the genome, we found a truncated mutation located in *mucA* of this strain, which was named *mucA117*. It can cause the production of mucus, reduce the expression of T3SS, and avoid the activation of inflammasomes. We speculate that this mutation favors the long-term presence of *P*. *aeruginosa* in the airway.

## Materials and Methods

### Bacterial strains and growth conditions

The bacterial strains used in this study are listed in Table 1. The *P*. *aeruginosa* strain PAO1 is the wild-type strain used in this study. *P*. *aeruginosa* strain L012 was isolated in 2021 from the sputum of a 74-year-old male bronchiectasis patient at Ruijin Hospital. All strains were grown on Luria-Bertani (LB) agar or in LB broth at 37ºC. LB supplemented with 5 mM EGTA and 20 mM MgCl _2_ was used as a T3SS inducing condition. Antibiotics were used at the following final concentrations: gentamicin (Gm) at 30 μg/mL, carbenicillin (Cb) at 150 μg/mL and tetracycline (Tc) at 100 μg/mL. In the strains containing the plasmids with an arabinose-inducible promoter, 10 mM arabinose was added to induce the expression of plasmid-borne genes. When observing the mucus formation of the strains, no additional arabinose was added.

**Table:** **Table 1** Bacterial strains and plasmids used in this study

Strain or plasmid	Description	Source or reference
*P*. *aeruginosa* strains		
PAO1	Wild-type strain	Stored in our laboratory
L012	Clinically isolated strain	Isolated from Ruijin Hospital
PAO1Δ *mucA*	PAO1 with *mucA* deleted; Gm ^r^	This study
Plasmids		
pCasPA	Bacterial expression of Cas9 nuclease and λ-Red recombination system; Tc ^r^	[1]
pACRISPR	A sgRNA expression plasmid for targeting a specific sequence; Cb ^r^	[1]
pACRISPR- *mucA*-spacer	pACRISPR derivative with the spacer of the *mucA*	This study
pACRISPR- *mucA*-spacer-Up-Gm-Downstream	pACRISPR derivative with the repair arms of the *mucA*	This study
pHERD20T (EV)	Shuttle vector with P _BAD_ promoter; Cb ^r^	From Dr. Yufeng Yao’s lab
pPAO1 *mucA*	PAO1 *mucA* carried on pHERD20T	This study
pL012 *mucA*	L012 *mucA* carried on pHERD20T	This study

Gm ^r^, gentamicin resistance; Tc ^r^, tetracycline resistance; Cb ^r^, carbenicillin resistance.

### Genome sequencing and assemblies

DNA extracted from the *P*. *aeruginosa* strain L012 was subjected to whole genome sequencing using Novoseq 6000 (Novogene, Beijing, China) following the supplier’s protocol (Illumina, San Diego, USA). Surviving reads were assembled by a *de novo* approach using SPAdes [20]. To perform the pan-genome analyses, genome sequences were first annotated with Prokka [21]. Pan-genomes were then analyzed with Roary [22], and Snippy was used for variant detection. For KEGG functional annotation and pathway analysis, BlastKoala [23] was used with mutated genes.

### Construction of deletion mutants and plasmids

The plasmids used in this work are listed in Table 1. The construction of the strain PAO1Δ *mucA* was as described in a previous study [24]. The primers used are shown in Supplementary Table S1. After pACRISPR was digested with *Bsa*Ι (NEB, Massachusetts, USA), the spacers were ligated to the plasmid. Then, the pACRISPR- *mucA*spacer was digested with *Xba*Ι (Thermo Scientific, Waltham, USA) and *Xho*Ι (Thermo Scientific). The 500 bp fragment upstream of the start codon, the 500 bp fragment downstream of the stop codon of *mucA* and the Gm fragment were amplified by PCR, and then these three fragments were ligated into the digested pACRISPR- *mucA*-spacer. pCasPA was electroporated into the *P*. *aeruginosa* strain PAO1, and then pACRISPR- *mucA*-spacer-Up-Gm-Downstream was electroporated into this strain. Successful deletions were verified by sequencing.

To complement the PAO1Δ *mucA* mutant, the coding regions of the *mucA* locus were PCR amplified from *P*. *aeruginosa* strain PAO1 genomic DNA using the primers listed in Supplementary Table S1, which were designed to include *Hin*dΙΙΙ (Takara, Kyoto, Japan) and *Eco*RΙ (Takara) sites. The amplified product was cloned into pHERD20T. The plasmid pPAO1 *mucA* was tested for the correct insert by sequencing. Then, pPAO1 *mucA* was electroporated into the *P*. *aeruginosa* strain PAO1Δ *mucA*, and this strain was named PAO1Δ *mucA*/PAO1 *mucA*. pPAO1 *mucA* was also electroporated into *P*. *aeruginosa* strain L012 to construct L012/PAO1 *mucA*. The coding regions of the L012 *mucA* locus were obtained by PCR amplification from *P*. *aeruginosa* strain L012 genomic DNA and then used a similar strategy to construct *P*. *aeruginosa* strain PAO1Δ *mucA*/L012 *mucA*. pHERD20T (empty vector, EV) was electroporated into the *P*. *aeruginosa* strains PAO1Δ *mucA* and L012 to construct the *P*. *aeruginosa* strains PAO1Δ *mucA*/EV and L012/EV.

### Cell culture and *in vitro* infection assay

Peritoneal macrophages (PMs) were harvested from C57BL/6 female mice after intraperitoneal injection of 1 mL of 5% thioglycollate broth, as previously described [25]. Lavage the peritoneal cavity with 10 mL of cold PBS followed by centrifugation at 200 *g* for 5 min to obtain cells. PMs were cultured in 6-well cell culture clusters overnight at 37ºC with 5% CO _2_ and then infected with mid-log phase *P*. *aeruginosa* cells. The supernatants were collected 6 hours postinfection (hpi) for LDH detection and 24 hpi for ELISA detection. The supernatants and cell lysates were collected 6 hpi for caspase-1 detection. All animal experiment procedures were approved by Animal Ethics Review Committee of Shanghai Jiao Tong University (project number A-2018-021).

### Cell viability assay

The supernatants of PMs were collected 6 hpi, and cell death was evaluated by detecting the amount of LDH released. LDH release was measured by using a CytoTox 96 Non-Radioactive Cytotoxicity Assay kit (Promega, Madison, USA). A microplate reader was used to detect the absorbance at 490 nm.

### Inflammasome activation assay

PMs were infected with *P*. *aeruginosa* for 6 h, and the supernatants were treated with methanol and chloroform with vortexing and centrifugation at 10,000 *g* for 5 min to obtain total proteins. Cell lysates were obtained by lysing PMs in RIPA buffer (Sangon Biotech, Shanghai, China). Equal amounts of total proteins extracted from supernatants and lysates of PMs were diluted in 5× SDS-PAGE loading buffer and boiled for 5 min. Proteins were separated on 12% polyacrylamide gels. Following electrophoretic transfer of protein onto PVDF membranes (Millipore, Billerica, USA), membranes were blocked in 5% skim milk. The membranes were incubated with mouse anti-caspase-1 monoclonal antibody (Adipogen, San Diego, USA) at 4ºC overnight. Then, the membranes were incubated with HRP-conjugated mouse antibody (Beyotime, Shanghai, China) for 1 h at room temperature, followed by visualization using a chemiluminescent substrate (Thermo Scientific).

### T3SS protein secretion assay

Overnight *P*. *aeruginosa* cultures were subcultured in fresh LB containing 5 mM EGTA and 20 mM MgCl _2_ and grown to log-phase (OD _600_=1.0). Whole-cell lysates were obtained by sonication, and the supernatants were obtained by centrifugation. Bacterial supernatants were treated with 15% trichloroacetic acid (TCA) at 4ºC overnight. The precipitated proteins were collected by centrifugation and washed with acetone. Equal amounts of protein samples were diluted in 5× SDS-PAGE loading buffer and boiled for 5 min. Proteins were separated on 12% polyacrylamide gels and transferred onto PVDF membranes. The membranes were incubated with rabbit anti-PcrV polyclonal antibody at 4ºC overnight. Rabbit anti-PcrV polyclonal antibody was prepared by our laboratory. The PcrV protein of the *P*. *aeruginosa* strain PAO1 was expressed in *E*. *coli* BL21, and then the rabbit was immunized subcutaneously with the purified protein in Freund’s complete adjuvant. The membranes were incubated with HRP-conjugated rabbit antibody (Sigma, St Louis, USA) for 1 h at room temperature, followed by visualization using a chemiluminescent substrate.

### Total RNA isolation and quantitative real-time PCR

Total bacterial RNA was isolated from planktonic cells. The planktonic cells were prepared from the log-phase cells subcultured at 37ºC in LB broth (OD _600_=0.5). The RNA samples were prepared using TRIzol LS Reagent (Thermo Scientific) and then reverse transcribed into cDNA. The reverse transcription reaction was conducted using a SuperScript ΙΙΙ First-Strand Synthesis SuperMix Kit (Thermo Scientific). Primer sequences are shown in Supplementary Table S1. Then, real-time quantitative PCR (qPCR) was performed using ChamQ Universal SYBR qPCR Master Mix (Vazyme, Nanjing, China). The 30S ribosomal protein gene *rpsL* was used as an internal control. The following thermal cycler conditions were used: 30 s at 95ºC, followed by 40 cycles of 10 s at 95ºC and 30 s at 60ºC.

### Statistical analysis

The results were analyzed by GraphPad Prism version 8, and statistical significance was evaluated by *t* test. Differences were considered to be significant at *P*<0.05.

## Results

### A truncated mutation occurs in the MucA protein of *P*. *aeruginosa* strain L012, which can transform the strain into mucoid

**Figure 1:**
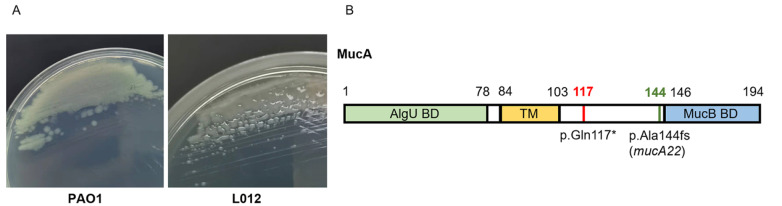
Figure 1 *P*. *aeruginosa* strain L012 is mucoid colonies and a truncated mutation of *mucA* occurs in strain L012 (A) P. aeruginosa strains PAO1 and L012 were grown on LB agar and imaged after 24 h to observe mucus formation. (B) Schematic diagram of the MucA structure. Green, AlgU binding domain (AlgU BD); yellow, the transmembrane domain (TM); and blue, the MucB binding domain (MucB BD). The red line indicates the mutation position of P. aeruginosa strain L012, and the green line indicates the mutation position of mucA22.

The *P*. *aeruginosa* strain L012 used in this study was isolated from the sputum of a bronchiectasis patient at Ruijin Hospital. We found that *P*. *aeruginosa* strain L012 was phenotypically mucoid on LB agar ( Figure 1A). To explore the adaptive changes in *P*. *aeruginosa* strain L012 during chronic infection, we performed comparative analysis of the genome between *P*. *aeruginosa* strain L012 and the laboratory strain PAO1. We screened the genes unique to *P*. *aeruginosa* strain PAO1 and the genes with insertions, deletions, frameshifts and early terminations in *P*. *aeruginosa* strain L012, and compared to *P*. *aeruginosa* strain PAO1. Compared with *P*. *aeruginosa* strain PAO1, the gene mutations of *P*. *aeruginosa* strain L012 occurred in the bacterial secretion system, quorum sensing system, biofilm formation, regulatory genes and so on ( Supplementary Table S2). Among them, a truncated mutation was found in the *mucA* of *P*. *aeruginosa* strain L012, c. 349C>T (p. Gln117*), which was different from common mutation sites. We named this mutation *mucA117* in our research. The most common mutation in CF and bronchiectasis is *mucA22* [26], known as a deletion of a G residue in a string of five G residues located between positions 429–433 of the *mucA* coding region [27]. MucA is a protein located in the cytoplasmic membrane with 194 residues and contains three domains. The N-terminal domain (residues 0–78) is the AlgU binding domain (AlgU BD), residues 84–103 are the transmembrane domain (TM) and the C-terminal domain (residues 146–194) is the MucB binding domain (MucB BD) [ 28, 29] ( Figure 1B). *mucA117* led to MucA with a truncated C-terminal periplasmic domain, which might make it unable to bind with the periplasmic MucB protein and cause the dysregulation of downstream genes, such as alginate synthesis-related genes.

### *mucA117* can lead the strain to mucoid and reduce pyroptosis and inflammasome activation of PMs

**Figure 2:**
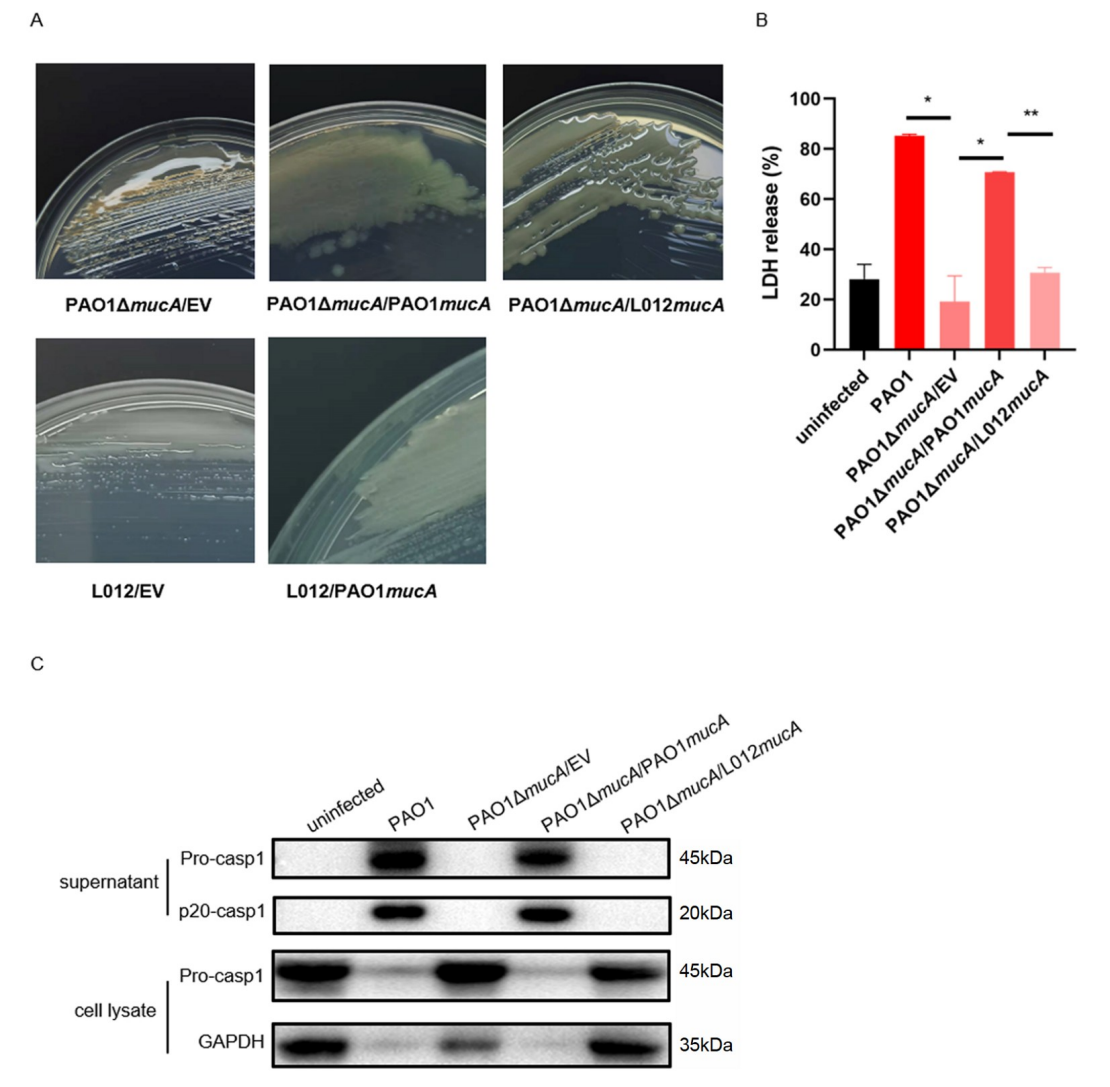
Figure 2 *mucA117* turns the strain into mucoid and reduces the its ability to induce pyroptosis and the activation of inflammasomes in PMs (A) The P. aeruginosa strains PAO1Δ mucA/EV, PAO1Δ mucA/PAO1 mucA, PAO1Δ mucA/L012 mucA, L012/EV and L012/PAO1 mucA were grown on LB agar without arabinose and imaged after 24 h to observe mucus formation. (B) PMs were infected with PAO1, PAO1Δ mucA/EV, PAO1Δ mucA/PAO1 mucA and PAO1Δ mucA/L012 mucA (MOI=10) for 6 h. Cell death was determined by measuring the LDH released into the supernatants. (C) Caspase-1 cleavage in the supernatants and cell lysates of PMs infected with PAO1, PAO1Δ mucA/EV, PAO1Δ mucA/PAO1 mucA and PAO1Δ mucA/L012 mucA for 6 h. * P<0.05, ** P<0.01 (unpaired t test).

We used the arabinose-inducible pHERD20T plasmid to express truncated L012 *mucA* in the *P*. *aeruginosa* strain PAO1Δ *mucA* (PAO1Δ *mucA*/L012 *mucA*) to explore whether *mucA117* has an effect on alginate synthesis. We found that after *mucA* was deleted, the strain changed from nonmucoid to mucoid. The PAO1Δ *mucA* mutant transformed into the nonmucoid phenotype after expressing wild-type *mucA* ( Figure 2A). However, the strain still maintained the mucoid phenotype after expressing L012 *mucA* ( Figure 2A). We also complemented wild-type *mucA* in *P*. *aeruginosa* strain L012 (L012/PAO1 *mucA*) with the pHERD20T plasmid. After expressing wild-type *mucA*, the *P*. *aeruginosa* strain L012/PAO1 *mucA* transformed into nonmucoid cells ( Figure 2A). These results showed that *mucA117* causes the strain to become mucoid.

*P*. *aeruginosa* strain PAO1 infection is able to activate NLRC4 and NLRP3 inflammasomes and initiate a type of rapid inflammatory cell death termed pyroptosis in macrophages [30]. PMs were infected with the *P*. *aeruginosa* strains PAO1, PAO1Δ *mucA*/EV, PAO1Δ *mucA*/PAO1 *mucA* and PAO1Δ *mucA*/L012 *mucA* to evaluate the ability to induce cell death, which was measured by the amount of LDH released into the supernatants. The amount of LDH released into the supernatants of cells infected with PAO1Δ *mucA*/EV and PAO1Δ *mucA*/L012 *mucA* was not significantly different from that in the uninfected group ( Figure 2B). However, they were both significantly lower than those in cells infected with the *P*. *aeruginosa* strain PAO1Δ *mucA*/PAO1 *mucA* ( Figure 2B). Then, we detected the activation of caspase-1. After PMs were infected with the *P*. *aeruginosa* strains PAO1 and PAO1Δ *mucA*/PAO1 *mucA*, caspase-1 was cleaved into the active form p20 ( Figure 2C). In contrast, caspase-1 cleavage could not be detected after infection with the *P*. *aeruginosa* strains PAO1Δ *mucA*/EV and PAO1Δ *mucA*/L012 *mucA* ( Figure 2C). These data indicated that both *mucA* deletion and *mucA117* will affect its function of inducing cell pyroptosis and activating inflammasomes.

We also infected PMs with *P*. *aeruginosa* strains L012, L012/EV and L012/PAO1 *mucA*. Unfortunately, neither L012/EV nor L012/PAO1 *mucA* induced cell death ( Supplementary Figure S1A) or inflammasome activation ( Supplementary Figure S1B) in PMs. We speculate that the reason why the *P*. *aeruginosa* strain L012 cannot cause inflammasome activation is complex and that *mucA* mutation may be one of them.

### *mucA117* downregulates the expression of T3SS and inflammasome ligand *fliC*, which may be related to the increased expression of *algU* and *algR*

**Figure 3:**
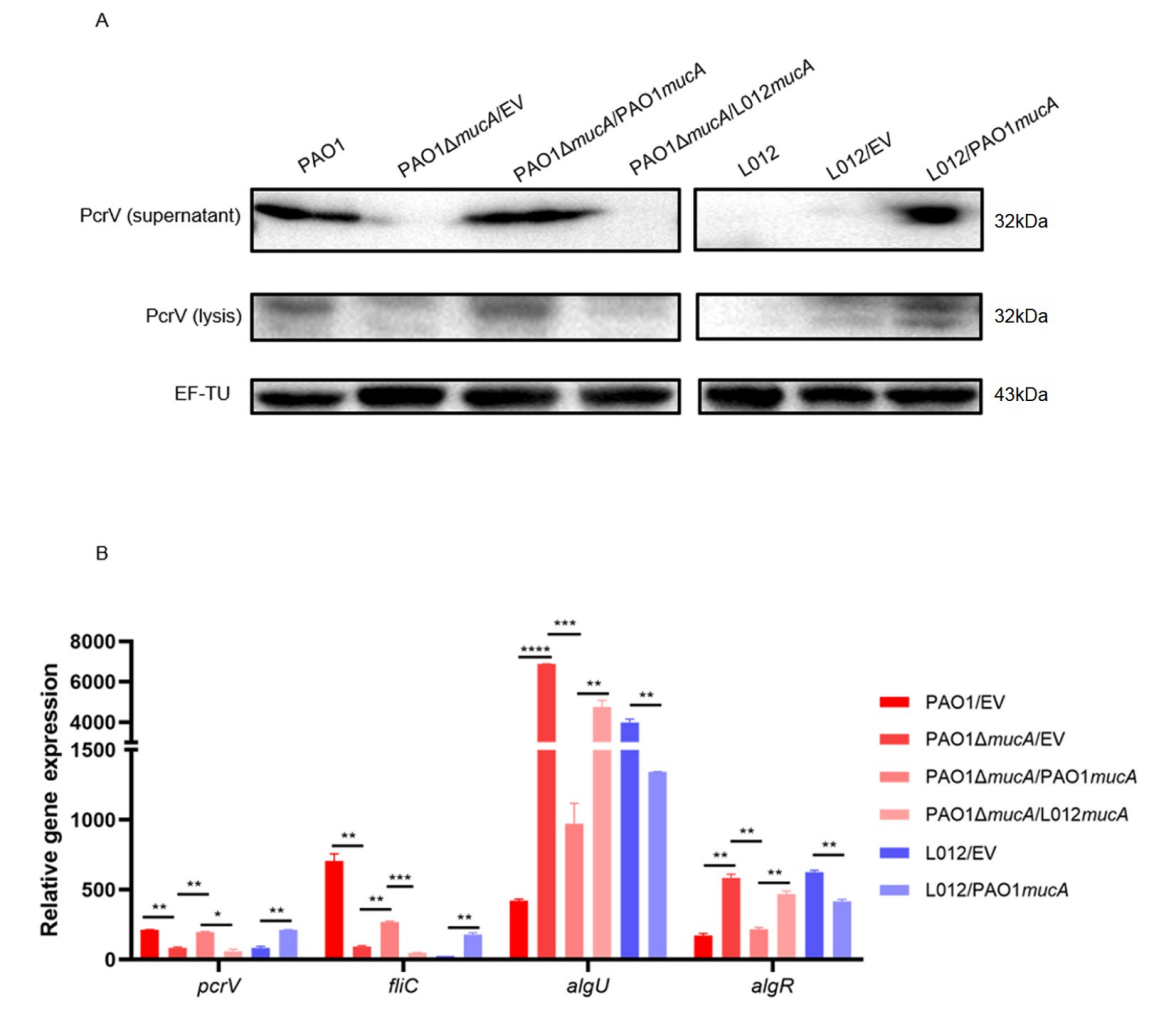
Figure 3 *mucA117* downregulates the expression of T3SS and inflammasome ligand, which may be related to the increased expressions of *algU* and *algR* (A) Under T3SS induction conditions, the PcrV levels in the P. aeruginosa strains PAO1, PAO1Δ mucA/EV, PAO1Δ mucA/PAO1 mucA, PAO1Δ mucA/L012 mucA, L012/EV and L012/PAO1 mucA were detected in culture supernatants and whole-cell extracts. (B) Relative expression of pcrV, fliC, algU and algR in these strains. * P<0.05, ** P<0.01, *** P<0.001, **** P<0.0001 (unpaired t test).

Transfer of inflammasome ligands into host cells via functional T3SS in *P*. *aeruginosa* is critical for inflammasome activation, and infection with T3SS-knockout *P*. *aeruginosa* cannot activate the inflammasome [31]. PcrV is an important structural translocation component which is necessary for T3SS secretion [32]. The protein FliC can be recognized by the NLRC4 inflammasome and secreted by T3SS [33]. As an important inflammasome ligand, it can induce inflammasome activation. In our study, we found that *mucA117* failed to induce pyroptosis or inflammasome activation in PMs. We speculated that this effect might be mediated by the reduced expression of T3SS and the key inflammasome ligands. Therefore, we detected the T3SS protein PcrV by western blot analysis and the mRNA expressions of *pcrV* and flagellin monomer *fliC* by qPCR analysis. As predicted, compared with those in the *P*. *aeruginosa* strains PAO1 and PAO1Δ *mucA*/PAO1 *mucA*, the T3SSs in the stains PAO1Δ *mucA*/EV and PAO1Δ *mucA*/L012 *mucA* were defective, and the expression of PcrV could not be detected in the whole-cell extracts or culture supernatants ( Figure 3A). Similarly, in the *P*. *aeruginosa* strains PAO1Δ *mucA*/EV and PAO1Δ *mucA*/L012 *mucA*, the expressions of *pcrV* and *fliC* were significantly lower than those in the *P*. *aeruginosa* strains PAO1/EV and PAO1Δ *mucA*/PAO1 *mucA* ( Figure 3B). These results showed that *mucA117* can downregulate the expression of T3SS protein and inflammasome ligands such as *fliC*, which might be the reason for avoiding the activation of inflammasomes and pyroptosis in PMs.

We also detected the expression of T3SS and inflammasome ligand *fliC* in *P*. *aeruginosa* strains L012/EV and L012/PAO1 *mucA*. After expressing wild-type *mucA* in L012, the ability of the strain to synthesize and secrete PcrV was restored ( Figure 3A). The relative expressions of *pcrV* and *fliC* were significantly higher in *P*. *aeruginosa* strains L012/EV and L012/PAO1 *mucA* than in L012/EV ( Figure 3B). The above results indicated that in *P*. *aeruginosa* strain L012, the decreased expressions of T3SS and inflammasome ligands are due to the truncated *mucA*. In *P*. *aeruginosa* strain L012, in addition to the truncated mutation of *mucA*, various types of mutations also occur in many important virulence genes, which may be the reason why *P*. *aeruginosa* strain L012/PAO1 *mucA* cannot induce pyroptosis or inflammasome activation in PMs.

Resent research shows that AlgU and the response regulator AlgR are required for *mucA*-mediated inhibition of T3SS gene expression [ 34‒ 36] . AlgU can also repress flagellum biosynthesis by inhibiting the expression of *fleQ* [37]. Therefore, we detected the relative expression of *algU* and *algR* in the strains by qPCR ( Figure 3B). In the *P*. *aeruginosa* strains PAO1Δ *mucA*/EV and PAO1Δ *mucA*/L012 *mucA*, the expressions of *algU* and *algR* were significantly higher than those in PAO1/EV and PAO1Δ *mucA*/PAO1 *mucA*. Consistent with these findings, the expressions of these two genes were significantly reduced in the *P*. *aeruginosa* strain L012/PAO1 *mucA*. Our results indicated that *mucA117* can increase the expressions of *algU* and *algR*, which is similar to a previous study on the *mucA22* mutant [ 34‒ 36] . We speculate that *mucA117* downregulates the expression of T3SS genes and flagellin gene *fliC* through increasing the products of *algU* and *algR*, thereby not activating the inflammasome.

## Discussion

A hallmark of *P*. *aeruginosa* in airways of chronic infections is their mucoid colony morphology, which results in the inactivation of MucA. The anti-sigma factor MucA is a transmembrane protein. Its N-terminal domain is located in the cytoplasm and can bind with AlgU, preventing the transcription of AlgU-dependent genes [38]. Its C-terminal domain is located in the periplasm and can bind with MucB, thereby preventing the cleavage of MucA by the periplasmic AlgW protease [39]. The loss-of-function mutation of *mucA* leads to increased transcription of *algU*. AlgU is an extracytoplasmic sigma factor (ECFσ) that can regulate at least 350 genes, including those responsible for the production of itself, *algR* and alginate biosynthetic enzymes [40]. The mutation types of *mucA* are very diverse. The most common mutation is *mucA22*, and this mutation makes the MucA protein unable to bind with the periplasmic MucB protein, making it less stable than wild-type MucA [41], which unbalances the MucA/AlgU ratio and leads to increased alginate synthesis [42]. In our research, we isolated *P*. *aeruginosa* strain L012 from a bronchiectasis patient and found a truncated mutation in *mucA* (p. Gln117*) named *mucA117*. Similar to the *mucA22* mutant, the MucA protein of *P*. *aeruginosa* strain L012 is truncated between the transmembrane domain and the periplasmic domain that interacts with MucB. Therefore, we speculate that the MucA of *P*. *aeruginosa* strain L012 cannot bind with the periplasmic MucB protein either, thereby releasing AlgU. As a result, *P*. *aeruginosa* strain L012 transforms into a mucoid colony.

Inflammasome activation occurs in response to a notably high number of pathogenic microbes and is critical for host defense against various microbial infections. During *P*. *aeruginosa* infection, the NLRC4 inflammasome is activated in response to recognition of bacterial flagellin by Naip5 [43], T3SS needle proteins EprI, MxiH, BsaL and PscF by murine Naip1 or by human NAIP (hNAIP) [44], and T3SS rod protein PscI by Naip2 [45]. Then, they trigger pyroptotic membrane rupture with subsequent secretion of IL-1β and IL-18. Recently, the lipid A motif of lipopolysaccharide (LPS) was shown to induce caspase-11-dependent pyroptosis [30]. When the inflammasome is activated, it can produce proinflammatory cytokines, allowing the recruitment of inflammatory and phagocytic cells to release proinflammatory mediators and elastase [46], which is necessary for bacterial clearance. However, this process also increases injury and pathological responses of the lung and is associated with poor host outcomes [47]. Current studies have shown that the expressions of T3SS genes, including the T3SS needle protein PscF, is downregulated in the *mucA22* mutant [34]. This regulation is AlgU- and AlgR-dependent. The study by Tart *et al*. [37] showed that the *mucA22* mutant can downregulate the expression of flagellin genes such as *fliC* through AlgU. Our results showed that *mucA117* led to increased expressions of AlgU and AlgR and downregulated the expressions of T3SS and inflammasome ligands, such as the flagellin monomer FliC. Therefore, we speculate that *mucA117* may have a similar function to the *mucA22* mutant in downregulating T3SS and flagellin genes, which might be a strategy to avoid inflammasome activation under selective pressure by the innate immune system during chronic infection. Failure to activate the inflammasome may make it difficult for the host’s immune system to clear *P*. *aeruginosa*, thereby allowing long-term colonization of the strain in the respiratory tract.

However, in the *P*. *aeruginosa* strain L012/PAO1 *mucA*, the expression of the T3SS protein PcrV and *fliC* was increased, and the expressions of *algU* and *algR* were decreased, but the ability to induce cell death and inflammasome activation of PMs was not restored. In the process of chronic infection, strains have undergone a rich and complex evolutionary process to adapt to the respiratory environment. In addition to the truncated mutation of *mucA*, various types of mutations also occur in other key virulence factors of *P*. *aeruginosa* strain L012. For example, we found a deletion mutation (p. Arg168_Leu169del) in the T3SS gene *pscK* and an insertion mutation (p. Pro4del insGlnAla) in the T3SS gene *pcrH*. PscK is a member of the SctK protein family, which can act as an adaptor protein interfacing SctQ with the 24-fold symmetric SctD inner membrane ring [48]. Its main function is to identify the secretion substrate and drive the secretion process. PcrH is the chaperone protein of PopB and PopD, which is responsible for the presecretory stabilization and efficient secretion of PopB and PopD translocators [49]. Compared with the wild-type strain, Δ *pcrH* cannot induce morphological changes in monolayer HeLa cells because it cannot maintain effective PopB and PopD secretion and therefore cannot transfer cytotoxins [50]. The mutations in these genes may also lead to partial loss of T3SS function so that the inflammasome is not activated. We speculate that the attenuation of inflammasome activation may be caused by a combination of many factors, and *mucA117* is one of them, even though the expressions of the T3SS protein PcrV and some inflammasome ligands are increased.

In summary, we isolated a *P*. *aeruginosa* strain with a truncated mutation of *mucA* from a patient with bronchiectasis. This truncated MucA protein turned the strain into mucoid and downregulated the expression of T3SS and inflammasome ligands such as *fliC* and reduced inflammasome activation. These changes might make the strain difficult to clear, favoring its long-term colonization.

## Supplementary Data

Supplementary data is available at *Acta Biochimica et Biophysica Sinica* online.
